# Correction: Double cross-linked transparent superhydrophilic coating capable of anti-fogging even after abrasion and boiling

**DOI:** 10.1039/d3ra90080k

**Published:** 2023-08-25

**Authors:** Xingyu Liu, Lili Xu, Shuaisheng Zhao, Haoxuan Hua, Yifan Su, Xinquan Yu, Jinlei Wang, Gang Li, Youfa Zhang

**Affiliations:** a Jiangsu Key Laboratory of Advanced Metallic Materials, School of Materials Science and Engineering, Southeast University Southeast University Road Nanjing 211189 PR China 230228666@seu.edu.cn yfzhang@seu.edu.cn; b State Key Laboratory of Advanced Technology for Float Glass, CNBM Research Institute for Advanced Glass Materials Group Co., Ltd Bengbu 233000 PR China

## Abstract

Correction for ‘Double cross-linked transparent superhydrophilic coating capable of anti-fogging even after abrasion and boiling’ by Xingyu Liu *et al.*, *RSC Adv.*, 2023, **13**, 23409–23418, https://doi.org/10.1039/D3RA03113F.

The authors regret that the Author contributions statement was shown incorrectly in the original manuscript. The corrected Author contributions statement is shown here.

## Author contributions

Xingyu Liu and Lili Xu contributed equally to this work. Xingyu Liu: conceptualization, investigation, methodology, writing – original draft. Lili Xu: methodology, validation, conceptualization, theoretical analysis. Shuaisheng Zhao: investigation, validation, methodology, writing – review & editing. Haoxuan Hua: investigation. Gang Li: investigation. Jinlei Wang: investigation. Yifan Su: investigation. Xinquan Yu: funding acquisition, review & editing. Youfa Zhang: project administration, supervision, review & editing.

The Royal Society of Chemistry apologises for these errors and any consequent inconvenience to authors and readers.

## Supplementary Material

